# Supplementary material for: The phosphatidylinositol-5′ phosphatase synaptojanin1 limits integrin-mediated invasion of Staphylococcus aureus
Source: Microbiol Spectr. 2024 Feb 15;12(4):e02006-23. doi: 10.1128/spectrum.02006-23 (PMC10986543; doi:10.1128/spectrum.02006-23)
Supplement: Supplemental figures — Figures S1 and S2. [file spectrum.02006-23-s0001.pdf]

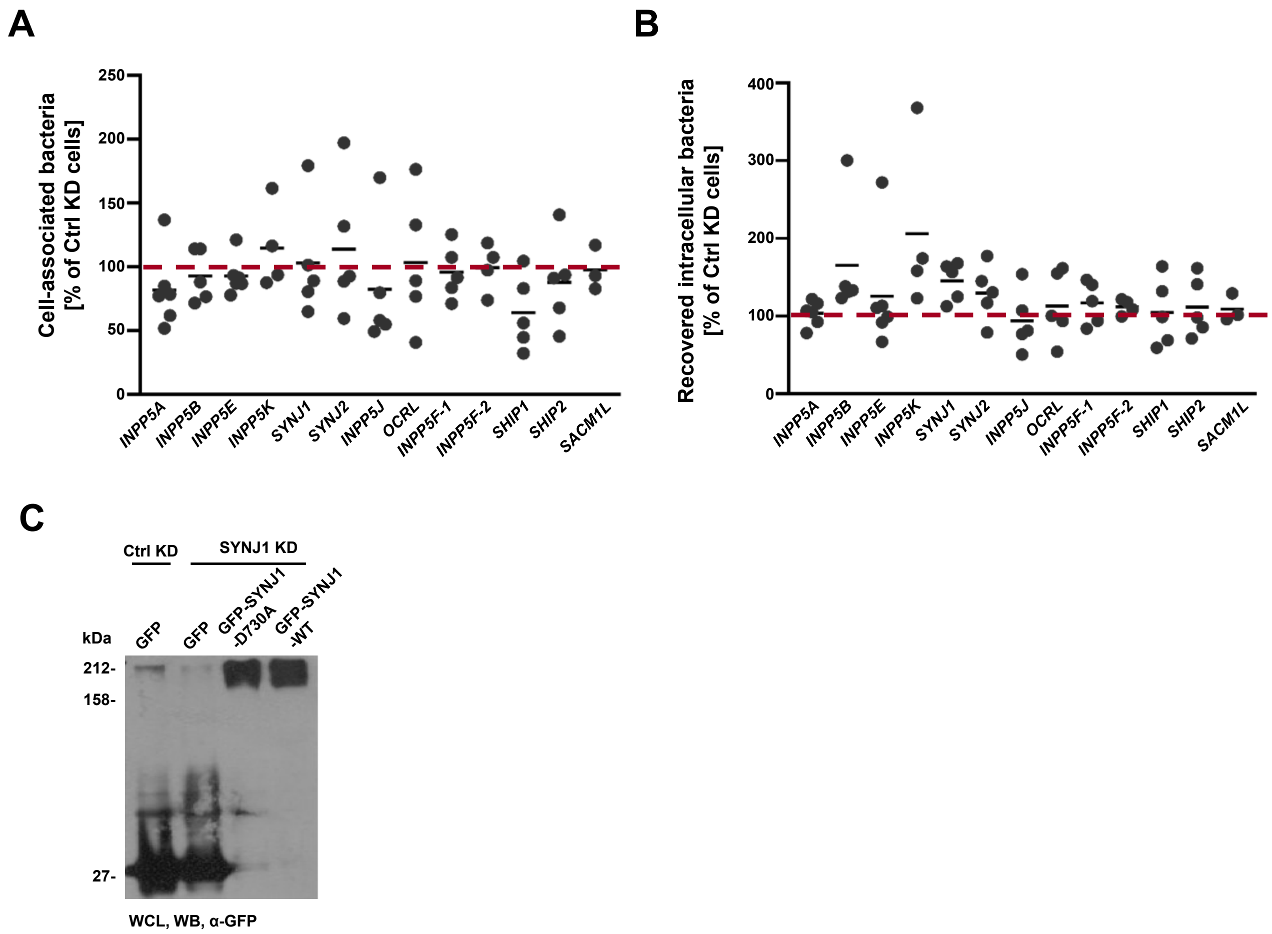

Suppl.Fig. S1 Knockdown of Synaptojanin-1 leads to increased uptake of *S. aureus*, which is reverted by re-expression of Synaptojanin-1.
(**A and B**) HEK293T cells with knockdown of individual 5'-phosphatases were infected with *S.aureus* for 2 hr. The total cell-associated (A) and the recovered intracellular bacteria (B) were quantified by gentamicin protection assays. The colony numbers obtained from control KD cells were set to 100, and used to normalize the values obtained from 5'-phosphatase KD cells. Each dot represents an independent assay, horizontal lines indicate mean values (n = 4-6). (**C**) Transient expression of GFP, GFP-SYNJ1-WT, or GFP-SYNJ1-D730A in Ctrl KD cells or SYNJ1 KD cells, respectively, was verified by Western blot with antibody against GFP.

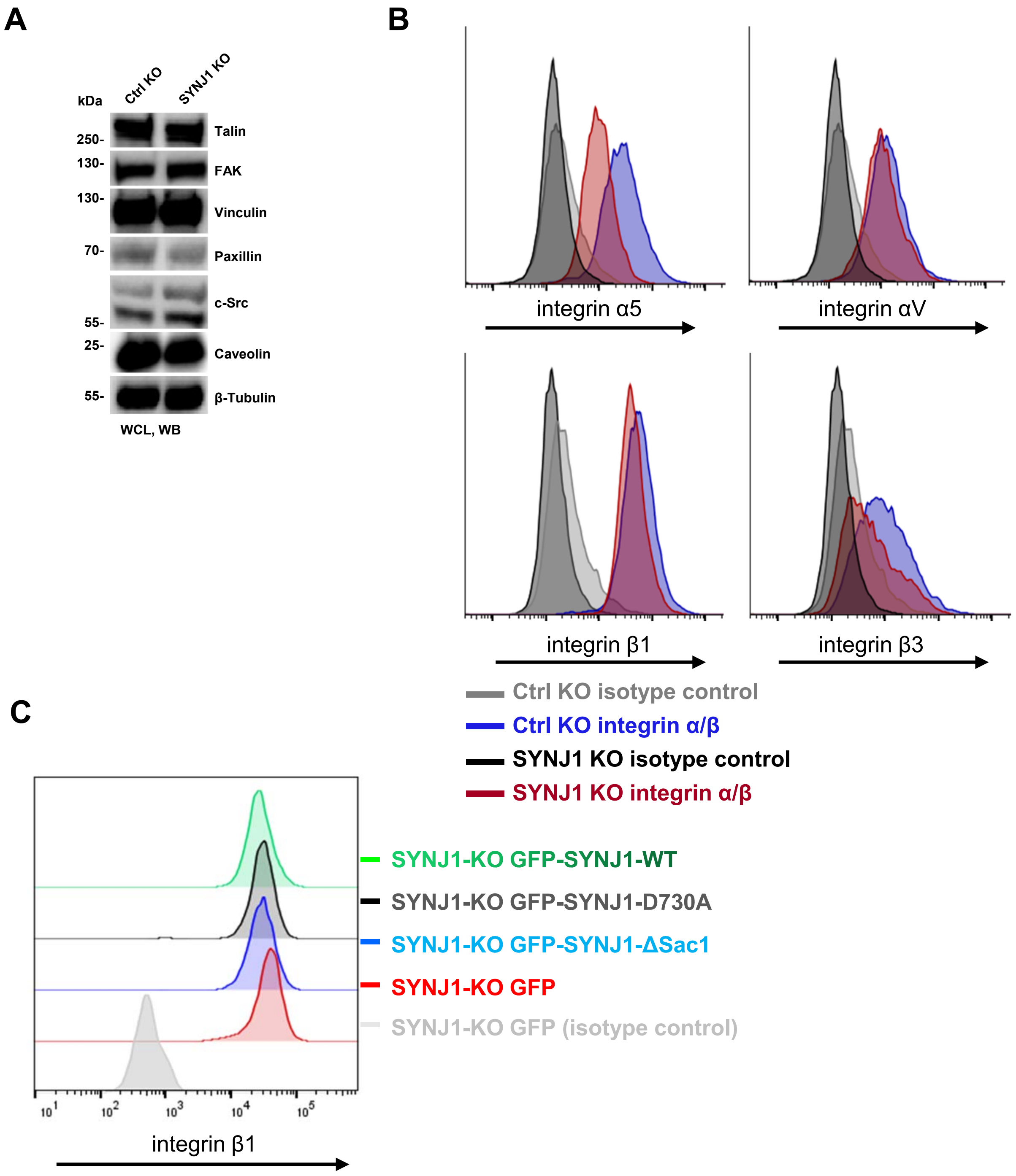

Suppl. Fig. S2 Cell surface level of fibronectin-binding integrins α5β1 and αvβ3 is reduced in Synaptojanin-1 knockout cells
(A) The levels of core focal adhesion proteins in whole cell lysates of control KO or SYNJ1 KO cells were detected by Western Blot with indicated antibodies. **(B)** Cell surface integrin α5, integrin αv, integrin β1, and integrin β3 expression by control KO or SYNJ1 KO cells was analyzed with monoclonal antibodies by flow cytometry. A sample of Control KO and SYNJ1 KO cells was stained with an isotype-matched control antibody (isotype control). A representative FACS analysis is shown. **(C)** Cell surface integrin β1 levels of SYNJ1 KO cells with stable re-expression of GFP, SYNJ1-WT, SYNJ1-D730A, or SYNJ1-ΔSac1 were analyzed by flow cytometry as in (B). A representative FACS analysis is shown.
